# Community Case of Methicillin-resistant *Staphylococcus aureus* Infection

**DOI:** 10.3201/eid1201.050279

**Published:** 2006-01

**Authors:** Lee Nelson, Clive S. Cockram, Grace Lui, Rebecca Lam, Edman Lam, Raymond Lai, Margaret Ip

**Affiliations:** * Prince of Wales Hospital, Chinese University of Hong Kong, Hong Kong SAR, People's Republic of China

**Keywords:** MRSA, CA-MRSA, BORSA, community, Hong Kong, Staphylococcus aureus, letter

**To the Editor:** Community-associated methicillin-resistant *Staphylococcus aureus* (CA-MRSA) is an emerging infectious disease worldwide and is increasingly reported in Asia (*1*). We describe a community case of invasive MRSA infection, which appeared as bacteremia and pneumonia; CA-MRSA was initially suspected, and eventually the patient was treated successfully with ampicillin/sulbactam.

A 52-year-old man with chronic eczema was admitted to the Prince of Wales Hospital, Hong Kong, with fever and chills. Before admission, he had been treated for infected eczematous lesions for several weeks with oral ampicillin, cloxacillin, and cefazolin. He had no history of hospitalization in the past 10 years, and none of his family members were healthcare workers. Examination showed an oral temperature of 40°C, blood pressure 95/55 mm Hg, and no audible murmur. Cellulitis in the left leg complicated his eczematous skin lesions. Chest radiograph showed right-middle-zone pneumonia. Neutrophilia (leukocytes 15.5 × 10^9^/L, neutrophils 86%), thrombocytopenia (platelets 55 × 10^9^/L), prolonged activated partial thromboplastin time (43.6 s), and elevated bilirubin level (31 μmol/L) were observed. Two initial blood cultures grew gram-positive cocci in clusters, identified as *S*. *aureus* by positive results for catalase and slide/tube coagulase and a negative result for ornithine decarboxylase. Intravenous cloxacillin (2 g every 6 h) was given on days 2–5. Antimicrobial drug susceptibility testing was performed by the disk-diffusion method (1 μg oxacillin/disk, Mueller-Hinton agar, 2% NaCl), followed by MIC determination with the agar dilution method in accordance with NCCLS (former National Committee for Clinical Laboratory Standards, now Clinical and Laboratory Standards Institute) recommendations (*2*). One blood isolate was identified as methicillin-resistant *S*. *aureus* (MRSA), with an oxacillin MIC 4 μg/mL. The other isolate was identified as methicillin-sensitive *S*. *aureus* (MSSA), with an oxacillin MIC of 0.5 μg/mL. In view of a possible CA-MRSA infection (which could have been β-lactam–resistant), cloxacillin was substituted with intravenous vancomycin plus rifampin on day 5.

However, the patient's condition progressively deteriorated from day 2 to day 10 with persistent fever, chills, hypotension, and hemoptysis. A repeated chest radiograph showed small lung cavities with fluid, and a thoracic computed tomographic scan confirmed multiple lung abscesses. Results of an initial transthoracic echocardiograph were normal, but a subsequent transesophageal echocardiograph demonstrated tricuspid valve vegetation.

The MRSA isolate was susceptible to gentamicin, cotrimoxazole, erythromycin, ciprofloxacin, clindamycin, fusidic acid, tetracycline, chloramphenicol, vancomycin, and rifampin; a different pattern of multidrug-resistant MRSA isolates from that usually found in our facility (*2**,**3*). The isolate was also susceptible to ampicillin/sulbactam, with an equivalent breakpoint MIC <8/4 μg/mL by disk testing (*2*). Latex detection for PBP2a (Slidex MRSA-Detection, bioMérieux, Marcy l'Etoile, France) and polymerase chain reaction (PCR) detection for *mec*A were both negative, predicting nonresistance to oxacillin (*2**–**5*). A nitrocefin-disk test was positive for β-lactamases, and a 4-fold reduction in MIC was demonstrated in the presence of sulbactam (*6*). Panton-Valentine leukocidin (PVL) gene locus was not detected (*1*). Community-acquired BORSA (borderline oxacillin-resistant *S*. *aureus*), infective endocarditis, and lung abscesses were diagnosed. Intravenous ampicillin/sulbactam (3 g every 6 h) was given on day 10 with rifampin; vancomycin treatment was stopped. Defervescence occurred 3 days later, subsequent blood cultures became sterile, and radiographic changes gradually resolved. Ampicillin/sulbactam was given for 6 weeks without complication.

As this case suggests, BORSA can sometimes be confused with CA-MRSA because of similar clinical signs and symptoms and overlapping oxacillin MICs (2–8 μg/mL and 4–64 μg/mL, respectively) (*1**,**4**,**6*). Both pathogens can appear as community-acquired infections and may be related to previous antimicrobial drug usage (*6**,**7*). Although CA-MRSA has been associated with soft tissue infections and necrotizing pneumonia (*7**,**8*), MSSA or BORSA strains can also cause these diseases. Thus, in view of potentially different treatment options, when MRSA isolates (e.g., oxacillin MICs >4–8 μg/mL) are associated with community-acquired, serious infections (e.g., blood isolates) and are not multidrug resistant, one can consider *mec*A (or PBP2a) testing to delineate the resistance mechanism (Table). If *mec*A is present, further testing for PVL gene locus with or without staphylococcal chromosomal cassette *mec* (*SCCmec*) type IV can be performed to diagnose CA-MRSA; if *mec*A is not detected, further testing for BORSA may be indicated, and β-lactam therapy should be evaluated individually. If these pathogens are not differentiated and all are treated as CA-MRSA, a non–β-lactam antimicrobial drug, such as vancomycin, will be used (*1**,**4**,**7**,**8*). However, for serious and deep-seated *S. aureus* infections (e.g., bacteremia, endocarditis), vancomycin is inferior to β-lactam antimicrobial drugs, even when in vitro testing indicates susceptibility. Treatment failures have been encountered (*4*). Linezolid is a good alternative but limited by availability and cost, and clindamycin therapy can be associated with inducible resistance. For BORSA-associated infections, β-lactam antimicrobial drugs, including high-dose penicillinase-resistant penicillins (PRPs) (e.g., cloxacillin) or β-lactam/β-lactamase–inhibitor combinations (e.g., ampicillin/sulbactam) are regarded as treatments of choice (*4**,**6**,**9*).

**Table:** Comparison between methicillin-sensitive and methicillin-resistant strains of *Staphylococcus aureus*

	Strain and major resistance mechanism*			
	MSSA/penicillinase production	BORSA/novel methicillinase ± penicillinase hyperproduction	CA-MRSA/PBP alteration	HA-MRSA/PBP alteration
PBP2a detection (e.g., latex-agglutination method)	–	–	+	+

*mec*A gene detection (e.g., PCR method)	–	–	+ (SCC*mec* IVa)	+

PVL gene detection (PCR method)	Infrequent (<5%)	Data limited	Frequent (>66%–100%)	Infrequent (<5%)

Coresistance to non–β-lactam antimicrobial drugs	±	±	+	+++

Usual antimicrobial drugs to which MSSA is susceptible	PRP (e.g., cloxacillin), β-lactam/β-lactamase–inhibitor combinations (e.g., ampicillin /sulbactam); linezolid, vancomycin, erythromycin, clindamycin, trimethoprim-sulfamethoxazole, fluoroquinolones, rifampin, gentamicin, fusidic acid, tetracyclines	PRP (e.g., cloxacillin), β-lactam/β-lactamase–inhibitor combinations (e.g., ampicillin/sulbactam), other drugs to which MSSA is potentially susceptible	Vancomycin, linezolid, rifampin, gentamicin, trimethoprim-sulfamethoxazole, fusidic acid, tetracyclines, fluoroquinolone, clindamycin†	Vancomycin, linezolid; ± fusidic acid, rifampin, gentamicin, trimethoprim-sulfamethoxazole, fluoroquinolones‡

*MSSA, methicillin-susceptible *Staphylococcus aureus*; BORSA, borderline oxacillin-resistant *S*. *aureus*; MRSA, methicillin-resistant *S*. *aureus*; CA-MRSA, community-associated MRSA; HA-MRSA, hospital-associated MRSA; PBP, penicillin-binding protein; PCR, polymerase chain reaction; PVL, Panton-Valentine leukocidin; PRP, penicillinase-resistant penicillins; +, positive; –, negative. ±, occasionally present; +++, usually present. †Concern over inducible clindamycin resistance; also, macrolide resistance is common. ‡Fluoroquinolone resistance increasing.

BORSA initially described nonheteroresistant strains of *S*. *aureus* with oxacillin MIC <2 mg/L, which produce ample β-lactamases and are rendered fully susceptible to PRP by β-lactamase-inhibitors (*4**,**6*). Subsequent BORSA strains described have had higher oxacillin MICs (4–8 mg/L) (*4*). The proportion of BORSA among clinical isolates of *S*. *aureus* varies (1.4%–12.5%) but is usually ≈5% (*4**,**10*). A BORSA infection outbreak among dermatology patients with severe skin diseases has also been reported (*10*). Postulated resistance mechanisms include overproduction of conventional penicillinases, production of an inducible, plasmid-mediated, membrane-bound methicillinase, and in some cases, point mutations of penicillin-binding-proteins (*4*). The clinical importance of BORSA is unknown since early clinical/animal data suggest treatment efficacy of PRP (against strains with MIC <2 mg/L) (*4**,**6**,**9*). Whether BORSA with higher oxacillin MICs (4–8 mg/L) will respond equally well to PRP is less clear. Further studies into the treatment of BORSA, including pharmacokinetic considerations, are needed (*4*). However, high-dose β-lactam/β-lactamase inhibitor combinations (e.g., ampicillin/sulbactam), as shown in animal models, are at least as effective as PRP (*9*). In conclusion, our report suggests that *mec*A (or PBP2a) detection may help manage serious, community-acquired, non–multidrug-resistant MRSA infections because of the potential confusion between BORSA and CA-MRSA.

## References

[1] Vandenesch F, Naimi T, Enright MC, Lina G, Nimmo GR, Heffernan H, Community-acquired methicillin-resistant *Staphylococcus aureus* carrying Panton-Valentine leukocidin genes: worldwide emergence. Emerg Infect Dis. 2003;9:978–84. 12967497 10.3201/eid0908.030089 PMC3020611

[2] National Committee for Clinical Laboratory Standards. Performance standards for antimicrobial susceptibility testing. Fifteenth informational supplement. NCCLS document M100-S15. Wayne (PA): The Committee; 2005.

[3] Ip M, Lyon DJ, Chio F, Enright MC, Cheng AF. Characterization of isolates of methicillin-resistant *Staphylococcus aureus* from Hong Kong by phage typing, pulsed-field gel electrophoresis, and fluorescent amplified-fragment length polymorphism analysis. J Clin Microbiol. 2003;41:4980–5. 10.1128/JCM.41.11.4980-4985.2003 14605127 PMC262491

[4] Chambers HF. Methicillin resistance in staphylococci: molecular and biochemical basis and clinical implications. Clin Microbiol Rev. 1997;10:781–91. 9336672 10.1128/cmr.10.4.781 PMC172944

[5] Brown DF. Detection of methicillin/oxacillin resistance in staphylococci. J Antimicrob Chemother. 2001;48(Suppl 1):65–70. 10.1093/jac/48.suppl_1.65 11420338

[6] Varaldo PE. The "borderline methicillin-susceptible" *Staphylococcus aureus.* J Antimicrob Chemother. 1993;31:1–4. 10.1093/jac/31.1.1 8444653

[7] Naimi TS, LeDell KH, Como-Sabetti K, Borchardt SM, Boxrud DJ, Etienne J, Comparison of community- and health care-associated methicillin-resistant *Staphylococcus aureus* infection. JAMA. 2003;290:2976–84. 10.1001/jama.290.22.2976 14665659

[8] Francis JS, Doherty MC, Lopatin U, Johnson CP, Sinha G, Ross T, Severe community-onset pneumonia in healthy adults caused by methicillin-resistant *Staphylococcus aureus* carrying the Panton-Valentine leukocidin gene. Clin Infect Dis. 2005;40:100–7. 10.1086/427148 15614698

[9] Hirano L, Bayer AS. Beta-lactam–beta-lactamase-inhibitor combinations are active in experimental endocarditis caused by beta-lactamase–producing oxacillin-resistant staphylococci. Antimicrob Agents Chemother. 1991;35:685–90. 2069374 10.1128/aac.35.4.685 PMC245079

[10] Balslev U, Bremmelgaard A, Svejgaard E, Havstreym J, Westh H. An outbreak of borderline oxacillin-resistant *Staphylococcus aureus* (BORSA) in a dermatological unit. Microb Drug Resist. 2005;11:78–81. 10.1089/mdr.2005.11.78 15770100

